# Experiences of psychiatric nurses caring for mental healthcare users with a comorbid disorder

**DOI:** 10.4102/curationis.v45i1.2354

**Published:** 2022-12-05

**Authors:** Annikie V. Hobyane, Nompumelelo Ntshingila, Marie Poggenpoel

**Affiliations:** 1 Department of Nursing, Faculty of Health Sciences, University of Johannesburg, Doornfontein, South Africa

**Keywords:** caring, comorbid, HIV, lived experiences, mental healthcare users, psychiatric nurses, schizophrenia

## Abstract

**Background:**

Caring for mental healthcare users (MHCUs) with a comorbid disorder of human immunodeficiency virus (HIV) and schizophrenia has always been challenging and requires expertise, skill, intuition and empathy.

**Objectives:**

The objective of this study was to explore and describe the experiences of psychiatric nurses caring for MHCUs with a comorbid disorder of HIV and schizophrenia.

**Method:**

A qualitative, exploratory, descriptive and contextual research design was used. Eight participants were selected through purposive sampling for individual in-depth interviews to collect data. Thematic analysis was used to analyse data.

**Results:**

Three themes emerged from this study. The first theme is that the psychiatric nurses experienced deep frustration because they were capable but unable to manage MHCUs with HIV and schizophrenia because of poor infrastructure and other contributing barriers. The second theme identified that the psychiatric nurses experienced discrimination against MHCUs compromising their holistic recovery. Lastly, the psychiatric nurses identified the need for health care workers in general hospitals and communities and families of MHCUs with a comorbid disorder to be educated in mental health issues to ensure continuous medical care.

**Conclusion:**

The results of this study showed that psychiatric nurses became exhausted when trying to cope with difficult nursing situations. The challenges they faced had negative consequences for the mental health of the psychiatric nurses and compromised patient care.

**Contribution:**

This study adds knowledge to nursing practice, nursing education and nursing research by implementing recommendations to mitigate the challenges of psychiatric nurses caring for MHCUs with HIV and schizophrenia.

## Introduction

Mental disorders are extremely common among patients with human immunodeficiency virus infection and acquired immunodeficiency syndrome (HIV/AIDS); but despite this, they are frequently underdiagnosed or poorly treated (Jonsson et al. 2013:155). A comorbid disorder refers to the manifestation of two or more mental disorders in a patient simultaneously (Middleton 2020:443). In this study, a comorbid disorder refers to a mental healthcare user (MHCU) suffering from HIV and schizophrenia. The complexity and nature of a comorbid disorder, such as HIV and schizophrenia, influence the pathophysiology of various systems in the body of MHCUs (Mjacu 2015:1). Mental healthcare users with a comorbid disorder, such as schizophrenia and HIV, have an increased risk of experiencing poor physical health compared with the general population (Haddad et al. 2016:1). According to Dolder, Patterson and Jester (2004:S36), HIV often results in higher morbidity and mortality in patients with schizophrenia than in the general population because these patients are more likely to struggle with treatment compliance and have a harder time communicating their symptoms to medical professionals. Moreover, the impact and burden of HIV-related infection are strongly associated with increased mental disorders, such as schizophrenia (Middleton 2020:720). Compared with 13% of the general population, it is estimated that 26% – 38% of South Africans living with HIV have a mental illness (Jonsson et al. 2013:156). It is estimated that up to 15% of the HIV-positive population has schizophrenia; among those with schizophrenia, 2.6% – 59.3% are in sub-Saharan Africa (Jonsson et al. 2013:158).

Most MHCUs suffering from a comorbid disorder are admitted to psychiatric hospitals. Comorbidity poses a significant challenge for psychiatric nurses who work in mental healthcare settings and care for MHCUs because it is challenging to handle this population of patients given structural issues and a lack of specialised facilities on the psychiatric wards (Giandinoto & Edward 2014:1). Additionally, it makes it difficult for psychiatric nurses to oversee the treatment programmes of these MHCUs. Most MHCUs with a comorbid disorder such as HIV and schizophrenia lack insight into their condition, so they depend on psychiatric nurses for their daily grooming, treatment and care (Iliceto et al. 2013:1), thereby creating more challenges for psychiatric nurses. Caring for MHCUs who are physically weak and sometimes become bedridden in this dramatic environment creates challenges for psychiatric nurses. The link between HIV and mental disorders results in overcrowded psychiatric wards and creates challenges and changes in psychiatric nursing care (Sadock & Sadock 2015:373).

The researcher observed in her 30 years of experience that psychiatric nurses caring for MHCUs with this specific comorbid disorder in psychiatric hospitals face significant challenges. Mental healthcare users with a comorbid disorder generally require special care, such as feeding, bathing, intravenous infusion and oxygen therapy as part of their treatment plan when they are seriously physically ill. Moreover, their rights are often violated by fellow MHCUs. Therefore, the psychiatric ward is not a conducive or appropriate setting to care for MHCUs with a comorbid disorder (Kneisl & Trigoboff 2014:136).

The MHCUs with a comorbid disorder that require assistance from psychiatric nurses provide a challenge to them. Psychiatric nurses who care for MHCUs diagnosed with a comorbid disorder need urgent organisational support and training to overcome such obstacles (Giandinoto & Edward 2014:1). However, there is a gap in knowledge about psychiatric nurses’ lived experiences of caring for MHCUs with a comorbid disorder in the Limpopo province. Therefore, the research question that arose from this problem statement was as follows: What are psychiatric nurses’ lived experiences of caring for MHCUs with a comorbid disorder of HIV and schizophrenia in a psychiatric hospital in Limpopo province?

### Aim

The aim of this study was to explore and describe the lived experiences of psychiatric nurses caring for MHCUs with a comorbid disorder of HIV and schizophrenia.

## Methods

### Study design and methods

A qualitative, exploratory, descriptive and contextual research design (Gray, Grove & Sutherland 2017:356) was utilised in this study. Exploratory research is employed when the researcher has observed something and seeks to understand it more clearly (Gray et al. 2017:359). In this study, the lived experiences of psychiatric nurses caring for MHCUs with a comorbid disorder in a psychiatric hospital in Limpopo province were explored. Descriptive research design describes how certain life events impact participants’ experiences (Edmonds & Kennedy 2013:130). This study described the lived experiences of psychiatric nurses caring for MHCUs with a comorbid disorder in a psychiatric hospital in Limpopo province. This study was contextual as it focused on the lived experiences of psychiatric nurses caring for MHCUs with a comorbid disorder in a psychiatric hospital in Limpopo province.

### Setting

The research setting was a specialised psychiatric hospital in Limpopo province, Thohoyandou. This specialised psychiatric hospital serves the community of Vhembe district and surrounding areas. It is situated in a remote area without shops nearby, and the community’s primary mode of transport includes buses and taxis. The psychiatric hospital is a 250-bedded hospital; 133 MHCUs were admitted to the psychiatric hospital, and 11 were diagnosed with the specific comorbid disorder, namely schizophrenia and HIV, at the time of the study. The psychiatric hospital has 10 wards and one outpatient department. The range of services in this specialised psychiatric hospital included acute psychiatric services, forensic psychiatry, intellectual disability, geriatric psychiatry and chronic psychiatric services. There were 100 psychiatric nurses in the hospital at the time of data collection. The researcher applied to the Chief Executive Officer of the psychiatric hospital where the study was conducted for permission to conduct the study. The researcher was not employed at the facility where the study was conducted. The psychiatric hospital where the study was conducted was selected to avoid coercion as the researcher is an operational manager at her workplace in another psychiatric hospital in Limpopo province. The researcher had a meeting with the hospital management which gave the researcher time to meet with the psychiatric nurses. The researcher met with the psychiatric nurses, informed them about the study, and invited them to participate. When the psychiatric nurses decided to participate in the study, they gave the researcher their phone numbers. The research setting was the psychiatric hospital, but the interviews were conducted in the psychiatric nurses’ homes after working hours.

### Study population and sampling strategy

The participants selected from the target group consisted of 100 psychiatric nurses caring for MHCUs with a comorbid disorder admitted to a psychiatric hospital. Purposive sampling was used to select participants (Gray et al. 2017:345).

Participants had to meet the following inclusion criteria to take part in the study:

- They could be male or female psychiatric nurses. This was to ensure that all psychiatric nurses were included in the study.
- They had to care for MHCUs with a comorbid disorder of HIV and schizophrenia in a psychiatric hospital because this was the focus of the study. The aim of the study focused on psychiatric nurses caring for MHCUs with a comorbid disorder of HIV and schizophrenia.
- They had to be registered as psychiatric nurses with the South African Nursing Council.
- Had to have two or more years of experience in providing care to MHCUs with a comorbid disorder to ensure adequate experience in nursing practice.
- Participants preferably had to be able to communicate in English.
- Participants had to be willing to participate. This ensures that participants are not coerced to participate in the study.

This study’s exclusion criterion was psychiatric nurses caring for MHCUs with comorbid disorders without diagnoses of HIV and schizophrenia. The reason for this exclusion criterion was that the aim of the study focused on psychiatric nurses caring for MHCUs with a comorbid condition of HIV and schizophrenia.

### Data collection

The researcher conducted individual in-depth phenomenological interviews and observations, and used field notes to explore and describe the phenomenon. Moreover, interviews were audio-recorded to allow for improved data analysis (Reiners 2012:2). Data collection took place from 01 March 2021 to 31 March 2021. The interviews were conducted at participants’ homes in a quiet space. Participants’ consent was obtained to audio-record the interviews before the process of data collection commenced to explore psychiatric nurses’ lived experiences of caring for MHCUs with a comorbid disorder. The researcher asked one main open-ended question: ‘What is it like to care for MHCUs with a comorbid disorder of HIV and schizophrenia?’ Eight interviews were conducted in English and lasted 45–60 min. The researcher’s responses were minimal to give the participants more time to express their views. By using bracketing, the researcher mitigated the potentially harmful effects of preconceptions that may taint the research process. Bracketing was used so that the researchers’ subjectivity did not skew data collection, analysis and interpretation, and to block any preconceived notions that were known and might have influenced data collection from the participants (Neubauer, Witkop & Varpio 2019:93).

### Data analysis

Data analysis includes coding and reflecting on thought processes in assigning meaning to data. The researcher sought meaning from all raw data and used Tesch’s thematic data analysis method (Polit & Beck 2017:531) to analyse and make sense of the collected data. Units of meaning were identified and organised from the data, transcribed interviews and field notes, and were joined together to form themes with supporting categories. The raw data were also handed to an independent coder for analysis. The independent coder was purposely selected because of their experience in the qualitative research approach using the same protocol for data analysis. The researcher and the independent coder met for a consensus discussion on the results of the data analysis.

### Trustworthiness

Guba’s model of trustworthiness was adhered to; trustworthiness was confirmed when the findings provided rich descriptions of the lived experiences of psychiatric nurses caring for MHCUs with a comorbid disorder, as indicated by participants (Holloway & Galvin 2017:309). According to Lincoln and Guba (1985), measures of trustworthiness include credibility, transferability, dependability and conformability; these were applied throughout the research to ensure the quality of the study. To ensure credibility, the researcher used data triangulation. This included using different data collection methods like in-depth phenomenological interviews, observations and field notes. The researcher involved two supervisors and one independent coder in this study to maintain investigator triangulation. Transferability was ensured by describing the research setting thoroughly and richly, and also describing what transpired during the research interviews in detail. An independent coder reviewed data separately from the researcher, and they both reached consensus on the themes, promoting the study’s dependability. Confirmability was ensured by conducting research interviews until data saturation was reached and an audit trail was developed, which entailed a systemic collection of documentation.

### Ethical considerations

Ethical clearance was obtained from the Faculty of Health Science Research Ethics Committee from the University of Johannesburg (Reference number REC-682-2020), and the Higher Degrees Committee (Reference number HDC-01-41-2020). The National Health Research Database in Limpopo province, Department of Health, Limpopo province, as well as the institution where the study was conducted also provided approval prior to the study’s commencement.

Human participants were involved in the research and as such their rights were protected. Four principles were therefore considered in conducting this research, namely autonomy, non-maleficence, beneficence and justice. These principles were adhered to throughout the research process. Informed consent was obtained from all participants, participation was voluntary, participants remained anonymous, and they were protected from any harm. The researcher obtained informed consent. The researcher was neutral as she was not employed at this specialised psychiatric hospital in Limpopo province. All participants were treated fairly, and the study aimed to promote their interests. The recorded interviews, transcripts and reports were stored safely under lock and key. The data will be kept for two years after the study’s publication.

## Results

The researcher conducted eight interviews, and data saturation occurred during the eighth interview. The psychiatric nurses were between the ages of 29 and 55 years; one was a widow, one was unmarried and six were married. Six were female, and two were male. They were all black South Africans, registered with the South African Nursing Council as psychiatric nurses and employed for a period of more than two years in the psychiatric hospital in Limpopo province at the time of data collection. See Table 1 for more details regarding the participants’ demographics.

**TABLE 1:** Participant demographics.

Participant number	Gender	Marital status	Age (years)	Nursing experience (years)	Qualifications
1	Female	Married	53	30	Diploma in General Nursing and Psychiatry
2	Male	Single	29	8	Diploma in General Nursing, Psychiatric, Community and Midwifery
3	Male	Married	49	20	Diploma in General Nursing, Psychiatric, Community and Midwifery
4	Female	Widow	45	15	Diploma in General Nursing, Psychiatric, Community and Midwifery BCur in Education and Management
5	Female	Married	40	19	Diploma in General Nursing, Psychiatric, Community and Midwifery
6	Female	Married	55	23	Diploma in General Nursing, Psychiatric, Community and Midwifery Diploma in Nursing Management
7	Female	Married	49	24	Diploma in General Nursing, Psychiatric, Community and Midwifery
8	Female	Married	45	18	Diploma in General Nursing and Psychiatric Nursing

*Source*: Adapted from Hobyane, A.V., 2022, ‘Lived experience of psychiatric nurses caring for mental health care users with a comorbid disorder at a psychiatric hospital in Limpopo province’, Master’s thesis, University of Johannesburg, Johannesburg, viewed 14 November 2022, from https://ujcontent.uj.ac.za/esploro/outputs/graduate/Lived-experiences-of-psychiatric-nurses-caring/9913087407691#file-0.

Three themes emerged from this study. See the results of the analysed in-depth interviews summarised in Table 2 for more details.

**TABLE 2:** Themes of the lived experiences of psychiatric nurses caring for mental healthcare users with a comorbid disorder in a psychiatric hospital in Limpopo province.

Themes	Sub-themes
Theme 1: Participants experienced deep frustration in the sense that they were capable but unable to manage MHCUs with a comorbid disorder because of infrastructure and other contributing barriers	Shortage of staff Lack of support Lack of infrastructure Lack of resources. Lack of medical equipment MHCUs defaulting on their treatment
Theme 2: Participants experienced that discrimination against MHCUs compromised their holistic recovery	Inadequate professional skills. MHCUs are denied access to medical treatment A fear of psychotic patients Referring MHCUs back to psychiatric hospitals without attending to their medical condition The assumption that the psychiatric nurses did not want to care for their own patients
Theme 3: Participants identified the need for other healthcare workers in general hospitals, as well as communities and MHCUs’ families, to be educated on mental health issues to ensure continuous medical care	Regular workshops should be convened to update nursing staff about MHCUs and their specific needs and treatment, as well as to remove the stigma of psychiatric disorders Nursing staff at general hospitals should be educated about admission and transfer procedures of MHCUs with a comorbid disorder They offered to conduct home visits to guide families in taking care of an MHCU They offered to run community campaigns about mental health issues to remove the stigma transpiring in communities They recommended training the SAPS and securities in mental health They offered to organise family days to educate MHCUs’ families about their family members’ treatment for mental disorders Listen to and act on their needs for adequate infrastructure to offer suitable care for MHCUs with a comorbid disorder Support and appreciate them and their efforts Provide extra nursing staff who can provide quality nursing care

*Source*: Adapted from Hobyane, A.V., 2022, ‘Lived experience of psychiatric nurses caring for mental health care users with a comorbid disorder at a psychiatric hospital in Limpopo province’, Master’s thesis, University of Johannesburg, Johannesburg, viewed 14 November 2022, from https://ujcontent.uj.ac.za/esploro/outputs/graduate/Lived-experiences-of-psychiatric-nurses-caring/9913087407691#file-0.

MHCUs, mental healthcare users; SAPS, South African Police Service.

### Theme 1: Participants experienced deep frustration in the sense that they were capable but unable to manage mental healthcare users with a comorbid disorder because of infrastructure and other contributing barriers

When psychiatric nurses were interviewed, it was discovered that they developed emotional responses, which included frustration, burnout, feeling overwhelmed, angry and demotivated, as they were failing to provide quality care to MHCUs because of infrastructure problems (Hobyane 2022:44). Most participants struggled with bottled-up emotions because of the restricted situation at the hospital. The psychiatric nurses caring for MHCUs with a comorbid disorder felt intense frustration at being trained and skilled to provide patient care, but being unable to do so because of the issues of a lack of adequate infrastructure and limited staff. The participants explained:

‘Another challenge is a stress of nurses due to shortage of staff and this will lead to frustration and resulted in burnout. The shortage resulted because there are people, who are resigning, and some are dying but there is no replacement of such people, and then the other people are going on pension and the department is not replacing anyone for that it creates a burden to nursing staff who are still working in the public institution.’ (Participant 8, 43 years old, female)

‘Nursing same MHCUs with the same condition, and it causes burnout and frustration because sometimes you think as a nurse you are not doing enough for MHCUs.’ (Participant 6, 55 years old, female)

Some indicated that they felt overwhelmed being caught in the middle of their patients and the hospital set-up, having to do their job without support and sufficient resources. They said:

‘[…*Y*]ou know we get exhausted as the healthcare providers, we become frustrated and we develop anger burn out and all these other things, because we feel we are not supported well by the management, and we left alone to suffer.’ (Participant 1, 53 years old, female)

‘[… *A*]lso strengthen the staff support, because some of staffs are tired, and some are no longer interested, as there is lack of support from the management, they don’t support the staff, they end up being … what can I say this? They are tired due to lack of support. So I think if the management they can change the way of managing and give proper support to staff.’ (Participant 4, 45 years old, female)

Psychiatric nurses indicated how they took out their frustrations and anger on their own families, harming their closest relationships:

‘Relationship with our family members is no longer fine, because when we go home we are tired and we don’t have time to interact well with family members and we are splinting the anger to the family members.’ (Participant 8, 45 years old, female)

‘E … I become easily irritated and angry and yaa … I project my anger to any one next me.’ (Participant 3, 49 years old, male)

Psychiatric nurses also reported a shortage of isolation rooms to isolate patients with infectious diseases. As such, their ability to perform their professional nursing roles was affected.

One participant explained the challenges of mixing patients:

‘There were no single rooms in which to isolate patients with infectious diseases, therefore patients were mixed, causing various challenges and the MHCUs with infectious disease meanwhile they should be separated from each other.’ (Participant 1, 53 years old, female)

Other participants mentioned the lack of a sickbay and reported poor disease control and prevention as there were no side wards to nurse MHCUs with infectious diseases. A participant explained how difficult it is to control cross infection:

‘So we don’t have a sick bay or isolation room where will nurse our physically ill MHCU though they are also mentally ill.’ (Participant 6, 55 years old, female)

‘We are nursing the very ill MHCUs in the midst of these other and they are sitting next to each other, it is difficult to control the spread of infection.’ (Participant 3, 45 years old, male)

One participant similarly described the causes of high infection rates at the hospital by stating:

‘Now during this Covid-19 [*coronavirus disease 2019*] pandemic is difficult for our patient to wear mask and is also difficult for them to wash their own hands so infection rate is high.’ (Participant 5, 40 years old, female)

Psychiatric nurses also experienced challenges related to a lack of specialised services such as X-rays, ophthalmic care and dental services, so patients needed to be transported to facilities with these services. Participants shared:

‘for an example like the patient a doctor may order the x-ray. At the hospital we don’t have that department. We have to transfer the patient to the nearest hospital to get that x-ray.’ (Participant 3, 49 years old, male)

‘We don’t have any … any … like x-ray and even when the patient has toothache we refer for consultation because we don’t have dental department in our hospital.’ (Participant 6, 55 years old, female)

In addition, participants reported a lack of suitable transport to take MHCUs to the general hospital for further management:

‘We are having an ambulance but is like taxi which you must get inside with a patient who is on oxygen, and there is no space to put that oxygen.’ (Participant 3, 49 years old, male)

‘The transport which we are using to transport the MHCUs to general hospital is not conducive as it seats all over because is a kombi and there is no space to put oxygen cylinder to administer to the MHCU while transporting him to the general hospital.’ (Participant 6, 55 years old, female)

The psychiatric nurses experienced a lack of medical supplies in all wards, such as oxygen, blood pressure (BP) monitors, suction machines and drip stands. As a result, they wasted valuable time walking up and down the hospital corridors borrowing necessary equipment.

Participants explained the shortage of medical supplies by stating:

‘There are no facilities to nurse the patient like BP monitor, oxygen, the sanction, all the apparatus that are used in general ward for observing the patient.’ (Participant 4, 45 years old, female)

‘Sometimes you find that the oxygen cylinder used as a resuscitation lifesaving equipment is not available in the ward you have to run around the whole hospital looking for the oxygen.’ (Participant 3, 49 years old, male)

In addition, psychiatric nurses experienced challenges with patients refusing to take their HIV medications and defaulting on their antipsychotic treatment at home. They consequently relapsed, causing burnout and frustration for the psychiatric nurses:

‘The MHCU when he is relapsed he become mentally unstable obvious he will not even comply with other treatment for HIV and he will become critically ill family members at home will brought the MHCU back to the hospital for readmission.’ (Participant 4, 45 years old, female)

‘[*B*]ut the difficulty comes when the patient is at home because he or she will be sometimes default treatment, and he/she will end up being relapsed.’ (Participant 5, 40 years old, female)

‘We re-admit the very same MHCUs with the same condition, and it causes burnout and frustration.’ (Participant 6, 55 years old, female)

### Theme 2: Participants experienced that discrimination against the mental healthcare users compromised their holistic recovery

According to the participants, nurses at general hospitals lacked skills in basic psychiatric nursing and needed to be trained to provide care to MHCUs admitted to the general hospital. Participant 4 explained this lack of knowledge about psychiatric patients as follows:

‘They lack knowledge on caring for psychiatric patients, or they just take it that this one is psychotic, he is pretending.’ (Participant 4, 45 years old, female)

Another participant indicated that general nurses’ lack of skills prompts them to discharge patients while they are still sick:

‘They don’t have skills most of them. So may be is the reason to make it difficult to care for him, so he will be discharged while still sick.’ (Participant 6, 55 years old, female)

Psychiatric nurses indicated that general nurses often denied MHCUs with a comorbid disorder access to medical treatment in general hospitals:

‘You might find that the MHCU come back while he is not yet healed due to the mental health status and stigmatisation.’ (Participant 3, 49 years old, male)

‘They deny them to access the service by sending them back to the hospital meanwhile they are fit for admission.’ (Participant 1, 53 years old, female)

Participants claimed that the staff at general hospitals refused to admit MHCUs with a comorbid disorder because they were scared of MHCUs, and patient safety was consequently compromised. Participant 2 stated that the following challenges lead staff in general wards not to admit MHCUs:

‘It is not easy for the nursing staff allocated at general hospital to receive the MHCU’s as they are afraid, and they don’t have skills….’ (Participant 2, 29 years old, male)

‘[*W*]e have challenge with that because, ya … like when we arrived there the nursing staff start to put stigma to these patients, because they are mentally ill, like the staffs at general hospital are scares of the mentally ill patient.’ (Participant 2, 29 years old, male)

Psychiatric nurses explained that a lack of awareness among staff at general hospitals led to the assumption that psychiatric nurses were forcing their MHCUs onto general hospitals.

‘When we transfer them to general hospital the staff there, they have negative attitude they will say “why do you bring the psyche patient” and they also sacred of psychiatric patient so it’s a problem.’ (Participant 4, 45 years old, female)

However, one participant explained that the shortage of equipment often forced them to transfer MHCUs to general hospitals:

‘We are transferring because, here we don’t have equipment for that, and is not like we are just pushing them to be at the medical ward or …’ (Participant 2, 29 years old, male)

### Theme 3: Participants identified the need for other healthcare workers, as well as communities and mental healthcare users’ families, to be educated on mental health issues to ensure continuous medical care

The psychiatric nurses indicated severe staff incompetency in providing quality care to critically ill MHCUs in psychiatric hospitals. They identified the need for regular workshops to be convened in order to update nursing staff about MHCUs’ conditions and specific needs, treatment, as well as to remove the stigma of psychiatric disorders.

Participants explained that in-service training should be provided to staff at general hospitals:

‘I think staffs at general hospital need in-service training on how to take care of mentally ill patient.’ (Participant 4, 49 years old, female)

‘Am suggesting that with the subsequent workshop or in-service training, they must also invite us to attend such workshop in order to capacitate us.’ (Participant 5, 40 years old, female)

The psychiatric nurses reported that the nursing staff at general hospitals need to be educated about the admission and transfer procedures of MHCUs with a comorbid disorder in order to provide quality care to MHCUs with a comorbid disorder when they are admitted to the general hospital.

One participant explained the benefit of developing staff’s capacity at the general hospital by stating:

‘I think they need to be capacitated in mental health issues in order for them to provide quality care even to patient, who are mentally challenged.’ (Participant 8, 45 years old, female)

Participant 6 also mentioned how the staff could further their studies through psychiatric courses:

‘[*I*]f they don’t have psyche course, we will just give them information about mental illness and, to encourage them to apply for study leave to go to the university or colleges furthering their studies on psychiatric courses.’ (Participant 6, 55 years old, female)

The psychiatric nurses offered to conduct home visits for families with relatives with a comorbid disorder in order to educate them on how to care for their loved ones. Participants also explained how to help the family members to manage mentally ill individuals at home:

‘[*W*]hat I suggest it may be home visit, if can do home visit to teach the family members about the management of mentally challenged individual so that they can know the effect of treatment on the psychiatric patient so that they can help MHCU when he is having a problem.’ (Participant 7, 49 years old, female)

Participants mentioned talking to parents of mentally ill individuals to assist their family members in continuing their treatment at home:

‘So we do try to talk to parents about how to assist the patient to take treatment at home.’ (Participant 5, 40 years old, female)

The psychiatric nurses offered to run community campaigns about mental health issues to remove the stigma transpiring in the communities.

‘I forget but awareness in the community just to talk about the mental illness and treatment of this mentally ill patient I think can also help.’ (Participant 6, 50 years old, female)

‘If we can run awareness campaign, and involve the community and the family members.’ (Participant 1, 53 years old, female)

Psychiatric nurses caring for MHCUs with a comorbid disorder said there is a need to train the South African Police Services in basic mental health as they often bring MHCUs to the hospital. One participant shared the particular need to train police staff in managing aggressive MHCUs:

‘Police staffs within the community are reluctant to attend to manage the aggressive MHCUs. I think they need training so that they will help us to manage the aggressive MHCUs.’ (Participant 1, 53 years old, female)

Another participant indicated the need to train police officers on general mental health issues:

‘And then training of the South African Police Services (SAPS) giving them information, basic information with regard to mental health.’ (Participant 3, 49 years old, male)

The psychiatric nurses offered to organise family days to equip MHCUs’ families with information about their relative’s treatment for mental disorders. Participant 7 also supported the idea of conducting a family day in order to observe the interactions between families and the MHCUs:

‘We also conduct family day where we invite the family members to come to the hospital and they stay we their siblings while observing them on how they interact with them before granting them LOA.’ (Participant 7, 49 years old, female)

One participant shared how and where a family day should be conducted:

‘To manage these MHCUs well, we also involve family members whereby we do invite parents to come and celebrate what we call family day in our institution.’ (Participant 5, 40 years old, female)

One participant spoke about the government’s involvement in building a new hospital for MHCUs with a comorbid disorder:

‘[*I*]f the department can help us building a new hospital I mean a new hospital which can cater for all MHCUs with a comorbid condition.’ (Participant 5, 40 years old, female)

Psychiatric nurses identified the need for management to appreciate the staff and their efforts. They should be supported to cope with their challenging job. A participant suggested the need for management to send staff for counselling and debriefing:

‘I suggest if the management can send us for counselling and debriefing so that we can relate our stories.’ (Participant 7, 49 years old, female)

Another participant indicated the need to be motivated to do good work by receiving tokens of appreciation:

‘[*A*]nd the another thing is that we also deserve to get token of appreciation in order to motivate us to continue doing the good work at all times.’ (Participant 8, 45 years old, female)

The psychiatric nurses need management to address the issue of staff shortages to provide quality care to MHCUs admitted within the psychiatric hospital. Ultimately, quality care reduces the cost and patients’ average length of stay within the psychiatric hospital.

A participant reported the need to hire more staff as some MHCUs need close monitoring:

‘He need close monitoring, meanwhile we don’t have enough staff to do that. The department must make sure that more nursing staff are hired.’ (Participant 2, 29 years old, male)

Another participant similarly indicated the need for government to hire more staff to render quality care to MHCUs:

‘It’s also a problem. So I think the management if they can add or the government can add enough staff to provide quality patient care.’ (Participant 1, 53 years old, female)

## Discussion

The purpose of this study was to explore and describe the lived experiences of psychiatric nurses caring for MHCUs with a comorbid disorder at a psychiatric hospital in Limpopo province. Three themes emerged from this study. The first theme was that the psychiatric nurses experienced deep frustration because they were capable but unable to manage MHCUs with a comorbid disorder of HIV and schizophrenia because of poor infrastructure and other contributing barriers. The second theme identified by this study is that the psychiatric nurses experienced discrimination against MHCUs compromising their holistic recovery. Lastly, the psychiatric nurses identified the need for other healthcare workers in general hospitals and communities and families of MHCUs with a comorbid disorder to be educated in mental health issues to ensure continuous medical care. The participants in this study expressed the negative consequences they experienced as ‘burnout’, which affects the care provided to MHCUs. Nakakis and Ouzouni (2020:189) indicated that the most frequently reported barriers to medical treatment were psychiatric nurses experiencing burnout because of inadequate support and limited job satisfaction.

Other participants were reportedly exhausted from trying to cope with tough nursing situations. These challenges had negative consequences on their mental health and compromised patient care. In support of this study findings, Cetrano et al (2017:2) suggest that high levels of fatigue overwhelm professionals’ sense of efficacy and prevent them from experiencing compassion satisfaction. (Cetrano et al. 2017:2). In addition, psychiatric nurses were often exposed to various attacks from MHCUs. According to Sim, Ahn and Hwang (2020:10), psychiatric nurses are constantly exposed to repeated, unexpected physical violence, and such incidents cause psychiatric nurses to become angry and act violently towards their patients.

Participants expressed being overworked because of insufficient staff availability, which had an emotional and physical impact on them. Engetou (2017:190) similarly reported that insufficient personnel leads to poor production. Caring for MHCUs with a comorbid disorder imposed stress on already strained psychiatric nurses.

Moreover, psychiatric nurses described the environment in which they care for MHCUs as challenging. Supported by research conducted in Jordan, Rola et al. (2017:8) indicated that the psychiatric hospital can be a challenging environment to work in, causing nurses to work under compromised conditions.

Psychiatric nurses indicated that stigmatisation has an impact on MHCUs’ access to quality care. According to Knaak, Manthler and Szeto (2017:2), several issues contribute to stigmatisation in healthcare and have a direct and indirect impact on access and the quality of care being provided for people living with mental illness.

Another challenge that psychiatric nurses experienced when caring for MHCUs with a comorbid disorder in a psychiatric hospital was a lack of resources. Moyimane (2017:325) supported the fact that critical shortage of medical equipment used by nurses to prevent, diagnose, and treat disease and promote rehabilitation created a barrier to the health system’s ability to deliver health services to MHCUs. In this study, a lack of equipment similarly hindered patients’ access to holistic healthcare.

The participants also experienced the phenomenon of MHCUs being violent towards each other, as confirmed by a study conducted by Caruso et al. (2021:2). Violence and aggressive behaviour are common among patients with mental illness admitted to psychiatric hospitals.

The participants of this study further described poor disease control and prevention practices. In support, Manyisa and Van Aswergen (2017:36) indicated that public hospitals exhibited numerous shortcomings, such as poor-quality service delivery for MHCUs with a comorbid disorder. Facilities are old, have poorly maintained infrastructure, and poor disease control and prevention practices.

The participants indicated that the MHCUs relapsed because of poor compliance with antipsychotic drugs. The same was reported in the study conducted by Olivares et al. (2013:1), who found that patients with schizophrenia often require antipsychotic drugs throughout their lives, and noncompliance typically drives relapse. Consequently, psychiatric nurses described the importance of family involvement when caring for MHCUs admitted to a psychiatric hospital. Kertchok, Yunibhand and Chaiyawat (2011:38) define the nurse-family relationship ‘as the process through which psychiatric nurses help family members’ so that they can provide better care for their loved ones and take care of themselves.

Participants shared that a vast lack of knowledge in caring for MHCUs with a comorbid disorder seemed evident among the nursing staff of general hospitals, and these patients were often denied access to medical treatment in general hospitals. Setona, Sehularo and Mokgaola (2020:14) highlighted that most general nurses do not cope well when providing care to MHCUs because of a lack of skill. They also concluded that specialised knowledge and clinical skills are required to cope in undertaking the role of mental healthcare. According to Ali et al. (2013:1), it was found that MHCUs were treated unfairly or were discriminated against by general health services. There were accounts of negative staff attitudes and behaviour, and a failure in services making reasonable adjustments for MHCUs. In addition, psychiatric nurses reported that staff at general hospitals refused to admit MHCUs with a comorbid disorder because they were scared of MHCUs, and patient safety was consequently compromised. Fear leads to poor nurse–patient interaction, resulting in poor-quality care (Jacobs & Holmes 2011:13).

Psychiatric nurses indicated that stigmatisation affects MHCUs’ access to quality care. Eissa et al. (2020:2) claim that stigma is a component of the negative discrimination that people with mental illness experience every day. It blocks access to facilities created to help people with mental illness and reduces the quality of medical service.

Psychiatric nurses explained that a lack of awareness among staff at general hospitals led to the assumption that psychiatric nurses were forcing their MHCUs onto general hospitals. A study conducted by Knaak et al. (2017:2) further acknowledges the power of hidden beliefs and attitudes that can underlie stigma-related behaviour.

Participants identified a need for regular workshops to be convened in order to update nursing staff about MHCUs’ conditions and specific needs, treatment, as well as to remove the stigma of psychiatric disorders. Knaak and Szeto (2015:12) agreed anti-stigma workshops should be conducted with healthcare providers to improve healthcare providers’ attitudes towards persons with mental illnesses. Such workshops could result in quality patient care. Participants highlighted that nurses’ education could play an important role in MHCUs’ care and should be prioritised. Sibeko et al. (2018:2) supported this theme, indicating that the primary outcomes of these training programmes for healthcare workers include improved healthcare worker knowledge, a change in healthcare workers’ confidence in supporting MHCUs, and a change in attitudes towards mental illness. Training programmes also improve the quality of care for MHCUs.

Psychiatric nurses caring for MHCUs with a comorbid disorder indicated the need to train the South African Police Services in basic mental health as they often bring MHCUs to the hospital. According to Fulde and Preisz (2011:2), MHCUs frequently display aggressive and violent behaviour because of underlying psychiatric disorders. Non-drug management and some simple preparatory steps may help avert trouble or deal with difficult situations as they arise. Police were sometimes called to mitigate MHCU crises, so training for police on basic information regarding mental health is required.

The participants further offered to run community campaigns about mental health issues to remove the stigma transpiring in the communities. According to Baumann (2014:17), awareness campaigns play an important role in informing the general public and creating advocacy groups to ensure MHCUs with comorbid disorders receive appropriate care. The participants offered to organise family days to equip MHCUs’ families with information about their relative’s treatment for mental disorders. Thomas, Liu and Umberson (2017:1) indicated that one important aspect of family days is to take care of each individual member to function at their best because many people in modern society suffer from mental health issues, and family support is critical.

Psychiatric nurses identified a need for management to appreciate the staff and their efforts. Kertchok et al. (2011:38) agree that psychiatric nurses need adequate support and help to grow on a personal level while providing care to MHCUs. Their views are supported by Nakakis and Ouzouni (2020:195), who state that management should address the immediate and long-term needs of staff in psychiatric hospitals. Skilled management that can resolve the conflict between nurses and recognise nurses’ efforts is required.

In addition, the participants shared the challenges they experienced when caring for patients with a comorbid disorder in an inadequately set up psychiatric hospital with insufficient resources and limited staff. Molehabangwe, Sehularo and Pienaar (2018:9) similarly claimed that improper infrastructure was one of the great concerns in public institutions where MHCUs with comorbid disorders are admitted. They are not properly designed to accommodate MHCUs, and as a result, newly built or renovated institutions are needed to provide quality care to MHCUs with a comorbid disorder. Participants also suggested that management hire more nurses to render care to MHCUs. Voogt et al. (2016:100) support the statement that skilled managers should provide enough nursing personnel, supervision, organisation, and direction for the professional staff and MHCUs.

### Limitations

The limitations of this study are that the psychiatric hospital delayed giving the researcher approval to commence data collection; it took three months to receive their approval. The researcher also had more female than male participants, because men were reluctant to participate in the study; only two male nurses volunteered to participate. Researchers could attempt to use less intense and more impersonal data collection methods in the future, such as questionnaires, which might be less threatening for male participants because male psychiatric nurses play an important role in the psychiatric hospital. The research was conducted among psychiatric nurses only. Other nursing categories, such as nursing assistants and enrolled nurses who also play an important role in the care provided to HIV-positive MHCUs within the psychiatric hospital, were not included in the research. The research was conducted in a district where the researcher was not working, and it took a long time for the researcher to visit the participants because of the distance between the two districts. Future research could be conducted in the district in which the researcher is working.

### Implications and recommendations

Little research has been conducted on this topic. There is a significant need to continue nursing research to explore whether the recommendations provided in this study effectively improved the mental health of psychiatric nurses caring for MHCUs with a comorbid disorder within a psychiatric hospital. Furthermore, additional research should be conducted in different institutions on the same topic to determine the impact of caring for MHCUs with a comorbid disorder within various psychiatric hospitals. Hospital research committees should permit more nursing research on mental health nursing to improve mental healthcare in psychiatric hospitals. The nursing curriculum should focus on caring for frail MHCUs with a comorbid disorder in psychiatric hospitals. The study results indicate that caring for MHCUs with a comorbid disorder within the psychiatric hospital can have a profound impact on the psychiatric nurses. Recommendations for nursing practice should ensure that psychiatric nurses are considered as human beings with body, mind and spirit to support their mental health as human beings in constant interaction with their environment; physically, socially, spiritually and psychologically. Therefore, comprehensive approaches should be used to address all aspects of the internal and external environment of psychiatric nurses at all levels of interaction. Recommendations to facilitate the mental health of psychiatric nurses caring for MHCUs with a comorbid disorder of HIV and schizophrenia include emotional and physical support to prevent and manage bottled-up emotions, physical adaptations to the hospital, and the availability of suitable infrastructure to care for MHCUs with a comorbid disorder. A system should be in place at managerial level to ensure that psychiatric nurses are supported in the care of MHCUs with a comorbid disorder. The policy of care should provide guidelines and procedures to ensure that the concerns of mental healthcare workers regarding the care of MHCUs with a comorbid disorder are addressed. These concerns can be addressed in policy, such as the HIV Policy and the *Mental Health Care Act*.

## Conclusion

This study aimed to explore and describe the lived experiences of psychiatric nurses caring for MHCUs with a comorbid disorder in a psychiatric hospital in Limpopo province. The researcher observed that the psychiatric nurses in the psychiatric hospital seemed to be demotivated, experienced a lack of support, and were exposed to various attacks from MHCUs. Psychiatric nurses described caring for MHCUs with a comorbid disorder within the psychiatric hospital as a ‘challenging experience’, resulting in a sense of failure in their mission as nurses because of being overburdened by multiple challenges. Participants were reportedly exhausted trying to cope with tough nursing situations, and their stress spills over from work to home; they desperately need to be heard. These challenges had negative consequences on the mental health of the psychiatric nurses and compromised patient care. The participants expressed the negative consequences they experienced as ‘burnout’. Several needs were expressed by the participants to address these challenges. Psychiatric nurses also experienced challenges with MHCUs’ families, as families often disown MHCUs with a comorbid disorder because they are a burden to them. Psychiatric nurses expressed frustration towards the families showing poor support towards MHCUs with a comorbid disorder because they can play a crucial role in the MHCUs’ recovery. Other challenges that psychiatric nurses experienced when caring for MHCUs with a comorbid disorder included a lack of resources, inadequate management support, a lack of teamwork, and nursing different kinds of MHCUs together with the very ill MHCUs in the same environment.
